# Prevalence of Pediatric Ocular Trauma in Northern Iran; An Epidemiological Cross-Sectional Study

**DOI:** 10.30476/BEAT.2021.90773.1262

**Published:** 2022-01

**Authors:** Hanieh Ahmadi, Zahra Alizadeh, Samad Karkhah, Mohammad Javad Ghazanfari

**Affiliations:** 1 *Department of Ophthalmology, Faculty of Medicine, Mazandaran University of Medical Sciences, Sari, Iran*; 2 *Student Research Committee, Mazandaran University of Medical Sciences, Sari, Iran *; 3 *Department of Medical-Surgical Nursing, School of Nursing and Midwifery, Guilan University of Medical Sciences, Rasht, Iran *; 4 *Burn and Regenerative Medicine Research Center, Guilan University of Medical Sciences, Rasht, Iran*; 5 *Department of Medical-Surgical Nursing, School of Nursing and Midwifery, Kashan University of Medical Sciences, Kashan, Iran*

**Keywords:** Pediatric ocular trauma, Ocular trauma, Blunt trauma, Epidemiology, Iran

## Abstract

Ocular trauma is one of the most common causes of acquired blindness in children. The epidemiological parameters associated with ocular trauma vary in different populations, especially in children. The objective of this study was to assess the ocular trauma epidemiology in children less than 18 years of age. In this cross-sectional study, 145 children (under 18 years) with ocular trauma who referred to the emergency department of Bu-Ali-Sina Hospital in Sari, Iran were enrolled from November 2017 to January 2019. Of the participants, 57.9% were men, 70.4% had blunt trauma, 97.2% had a unilateral eye injury, and 54.5% had a right eye injury. The most risk factor for trauma was stationery (51.0%). Almost half of the patients (52.9%) had corneal injuries. The most trauma locations were at home (67.4%). Most patients (95.0%) had normal relative afferent pupillary defects. Blunt (52.6% vs. 47.4%) and penetrating (72.5% vs. 27.5%) traumas was higher in boys than girls (*p*=0.03). Most frequent part of eye injuries in blunt and penetrating traumas was related to the cornea (*P*=0.04). It seems that parents should have more supervision on children at home and give adequate education in using of stationery to school-age children by considering the results of present study.

## Introduction

Ocular trauma is one of the most common causes of acquired blindness in children [1, 2]. Based on previous evidence, 1.6, 2.3, and 19 million people have become blind, bilateral low vision, and unilateral blindness due to ocular trauma, respectively [1, 3, 4]. Pediatric ocular trauma includes a range of corneal surface erosion to corneal and scleral perforation [5] and is associated with high emotional stress and increases the economic burden on society [3, 6]. However, 90% of ocular traumas can be prevented with proper care and treatment [1, 7, 8], especially in children, which is the most preventable cause of blindness [9]. 

The epidemiological parameters associated with ocular trauma vary in different populations [1, 10], especially in children due to natural curiosity and playing with dangerous and sharp objects [2]. A study in the USA showed that 25.4% of ocular traumas occur in children less than 18 years of age [11]. Another study in South Korea showed that the highest incidence of ocular trauma was in the age group of 10-14 years and was higher in boys than girls [12]. Also, another study in Brazil showed that the most common causes of ocular trauma in children were wood, stone, bicycles, broken glass, and falls, respectively [13]. 

Although previous evidence has confirmed that ocular trauma is the most common cause of blindness in developing countries and Iran, few studies have been performed in these countries [14, 15], especially in children less than 18 years of age. Therefore, due to the differences in the incidence of ocular trauma in different populations [10], the objective of this study was to assess the epidemiology of ocular trauma in children less than 18 years of age.

In this cross-sectional study, 145 patients with ocular trauma less than 18 years of age who referred to the emergency department of Bu-Ali-Sina Hospital in Sari, Iran were enrolled from November 2017 to January 2019. All patients with ocular trauma less than 18 years were included. Patients with previous history of eye disease and dissatisfaction to participate in this study were excluded. We used data gathering form, including age, sex, parental education level, trauma mechanism, unilateral/bilateral, part of eye injury, location of trauma and agent trauma. After completing the checklist, eye examinations were conducted by an ophthalmologist, including visual acuity, Marcus Gunn (relative afferent pupillary defect), slit lamp, and posterior segment. This study was authorized by the Institutional Ethics Committee of Mazandaran University of Medical Sciences. Written informed consent was obtained from patients. SPSS software package (version 18.0, SPSS Inc., Chicago, IL, USA) was used for data analysis. Quantitative and qualitative variables were presented using mean (standard deviation) and number (percentage), respectively. Chi-square test was used to assess the relationship between study variables. Statistical significance was considered *p*<0.05.

In total, 145 children (under 18 years) with ocular trauma were enrolled. Of the participants, 57.9% were boys, 70.4% had blunt trauma, 97.2% had a unilateral eye injury, and 54.5% had a right eye injury. The most risk factors for trauma were stationery (51.0%). Almost half of the patients (52.9%) had corneal injuries. The most trauma locations were at home (67.4%). Most patients (95.0%) had normal relative afferent pupillary defects (Table 1). Blunt (52.6% vs. 47.4%) and penetrating (72.5% vs. 27.5%) traumas was higher in boys than girls (*p*=0.03). There was a significant relationship between the trauma mechanism and part of eye injury. The most frequent part of eye injuries in blunt and penetrating traumas was related to the cornea (*p*=0.04). The visual acuity of the eyes was higher in penetrating trauma compared to blunt trauma (*p*>0.05) (Table 2).

**Table 1:** Demographic, epidemiological and examinations characteristics of participants (N=145)

	**Participant**s
Age (y)	8.0 (SD=4.5)
Sex	145 (100)
Men	84 (57.9)
Women	61 (42.1)
Father’s education level	118 (100)
Less than a diploma	13 (11.0)
Diploma	41 (34.7)
Associate Degree	16 (13.6)
BSc	36 (30.5)
MSc	8 (6.8)
PhD	4 (3.4)
Mother’s education level	118 (100)
Illiterate	1 (0.8)
Less than a diploma	11 (9.3)
Diploma	48 (40.7)
Associate Degree	11 (9.3)
BSc	37 (31.4)
MSc	8 (6.8)
PhD	2 (1.7)
Trauma Mechanism	135 (100)
Blunt	95 (70.4)
Penetrating	40 (29.6)
Type of eye injury	143 (100)
Unilateral	139 (97.2)
Right eye	78 (54.5)
Left eye	61 (42.7)
Bilateral	4 (2.8)
Part of eye injury	136 (100)
Conjunctiva	36 (26.5)
Cornea	72 (52.9)
Sclera	7 (5.1)
Anterior chamber	6 (4.4)
Non-injury	3 (2.2)
Agent trauma	143 (100)
Stationery	73 (51.0)
Toys	42 (29.4)
Body parts (fists and fingers)	16 (11.2)
Knife	10 (7.0)
Glass	2 (1.4)
Acid	0 (0.0)
Location of trauma	141 (100)
School	17 (12.1)
Home	95 (67.4)
Street	24 (17.0)
Other	5 (3.5)
Marcus Gunn	120 (100)
Normal	114 (95.0)
Abnormal	6 (5.0)
Visual acuity	10.1 (SD=1.2)
Right eye	10.1 (SD=1.1)
Left eye	10.0 (SD=1.4)

Data are presented as number (percentage) and mean (standard deviation).

**Table 2.** The relationship between visual acuity with part of eye injury and trauma mechanism in the participants (N=145)

	**N**	**Participants**	***p*** ** value**
Visual acuity of the right eye			0.19
Conjunctiva	29	10.1 (SD=0.9)	
Cornea	60	10.0 (SD=1.6)	
Sclera	6	9.7 (SD=0.8)	
Anterior chamber	5	9.6 (SD=0.5)	
Non-injury	1	7.0 (SD=0.0)	
Other	10	10.5 (SD=0.5)	
Visual acuity of the left eye			0.17
Conjunctiva	29	10.3 (SD=0.4)	
Cornea	59	10.1 (SD=1.3)	
Sclera	6	9.8 (SD=0.4)	
Anterior chamber	5	9.0 (SD=1.4)	
Non-injury	1	10.0 (SD=0.0)	
Other	10	10.5 (SD=0.5)	
Visual acuity of the right eye			0.81
Blunt	78	10.0 (SD=1.2)	
Penetrating	32	10.1 (SD=1.3)	
Visual acuity of the left eye			0.34
Blunt	78	10.0 (SD=1.2)	
Penetrating	32	10.2 (SD=0.9)	

Data are presented as number and mean (standard deviation); *p*-value was obtained with Chi-square test.

This study evaluated the ocular trauma epidemiology in children under 18 years. In the present study, the most common type of eye injury was related to blunt trauma which was confirmed by previous studies in South Korea [16] and Australia [17]. However, the results of a study in India [9] showed that penetrating traumas were the most common ocular trauma in children. These contradictions can be due to differences in the age group, study samples, methodology, and type and mechanism of injury [15].

In the present study, blunt and penetrating traumas were higher in boys than girls. These findings were consistent with studies in USA [18] and Iran [7]. Due to the aggressive nature of boys’ games, they are more exposed to eye injuries than girls [15]. 

In the present study, most injuries occurred at home. This finding was confirmed by various studies from Brazil [13], Lithuania [2], and New Zealand [19]. It can be justified by leaving children alone at home without parental supervision, which is a matter of concern [6, 7]. Most important risk factor for ocular trauma in children in the present study was stationery. Inconsistent with this finding, a study in China showed that the most common risk factor associated with ocular trauma in children was fireworks [3]. Also, a study in India reported that the most common risk factor was sports injuries [5]. These contradictions can be due to differences of the children’s age, culture, nationality, geographical location, and customs in different societies [8, 18].

Cornea was the most common site of eye injury in blunt and penetrating ocular trauma, which was supported by a study in India [5]. The pattern of ocular injury can be useful to elucidate the mechanism, clinical features, and preventive measures in pediatric ocular trauma [20]. The main limitation of this study was the difficult management of children with ocular trauma, including questionable circumstances surrounding ocular trauma and difficulties in collecting data from the ophthalmic examination.

In conclusion, this study showed that most children with ocular injury had blunt trauma at home. On the other hand, the most important risk factor for trauma in children less than 18 was the stationery. Therefore, parents should have more supervision on children at home and give adequate education in using of stationery to school-age children. 

## Declaration

### Ethics approval and consent to participate:

This study was authorized by the Institutional Ethics Committee of Mazandaran University of Medical Sciences. Written informed consent was obtained from patients. 

### Consent for publication:

Consent was obtained from the patients regarding the publication of this article.

### Conflict of interests

There are no conflicts of interest.

### Funding

There is no funding support for this study

### Authors’ contributions

1. All authors made a substantial contribution to the concept or design of the work; or acquisition, analysis, or interpretation of data.

2. All authors drafted the article or revised it critically for important intellectual content.

3. All authors approved the version to be published.

4. All authors have participated sufficiently in the work to take public responsibility for appropriate portions of the content.

### Acknowledgements

None.
